# *ant(6)-I* Genes Encoding Aminoglycoside O-Nucleotidyltransferases Are Widely Spread Among Streptomycin Resistant Strains of *Campylobacter jejuni* and *Campylobacter coli*

**DOI:** 10.3389/fmicb.2018.02515

**Published:** 2018-10-23

**Authors:** Lorena Hormeño, María Ugarte-Ruiz, Gonzalo Palomo, Carmen Borge, Diego Florez-Cuadrado, Santiago Vadillo, Segundo Píriz, Lucas Domínguez, Maria J. Campos, Alberto Quesada

**Affiliations:** ^1^Departamento de Bioquímica, Biología Molecular y Genética, Facultad de Veterinaria, Universidad de Extremadura, Cáceres, Spain; ^2^Centro de Vigilancia Sanitaria Veterinaria (VISAVET), Universidad Complutense Madrid, Madrid, Spain; ^3^Departamento de Sanidad Animal, Facultad de Veterinaria, Universidad de Extremadura, Cáceres, Spain; ^4^Departamento de Sanidad Animal, Facultad de Veterinaria, Universidad de Córdoba, Córdoba, Spain; ^5^MARE-Marine and Environmental Sciences Centre, ESTM, Instituto Politécnico de Leiria, Peniche, Portugal

**Keywords:** *Campylobacter coli*, Campylobacter jejuni, streptomycin-resistance, aminoglycoside adenylyl transferases, ANT(6)-I

## Abstract

Thermotolerant *Campylobacter* species *C. jejuni* and *C. coli* are actually recognized as the major bacterial agent responsible for food-transmitted gastroenteritis. The most effective antimicrobials against *Campylobacter* are macrolides and some, but not all aminoglycosides. Among these, susceptibility to streptomycin is reduced by mutations in the ribosomal RPSL protein or by expression of ANT(6)-I aminoglycoside O-nucleotidyltransferases. The presence of streptomycin resistance genes was evaluated among streptomycin-resistant *Campylobacter* isolated from humans and animals by using PCR with degenerated primers devised to distinguish *ant(6)-Ia*, *ant(6)-Ib* and other *ant*-like genes. Genes encoding ANT(6)-I enzymes were found in all possible combinations with a major fraction of the isolates carrying a previously described *ant*-like gene, distantly related and belonging to the new *ant(6)-I* sub-family *ant(6)-Ie*. Among *Campylobacter* isolates, *ant(6)-Ie* was uniquely found functional in *C. coli*, as shown by gene transfer and phenotype expression in *Escherichia coli*, unlike detected coding sequences in *C. jejuni* that were truncated by an internal frame shift associated to RPSL mutations in streptomycin resistant strains. The genetic relationships of *C. coli* isolates with ANT(6)-Ie revealed one cluster of strains presented in bovine and humans, suggesting a circulation pathway of *Campylobacter* strains by consuming contaminated calf meat by bacteria expressing this streptomycin resistance element.

## Introduction

Campylobacteriosis is the main cause of foodborne diseases in the UE and in the United States [Collective Eurosurveillance Editorial Team, 2015; (Accessed March 2018) ^1^]. The drugs of choice for the treatment of campylobacteriosis were, mainly erythromycin (ERY) and ciprofloxacin (CIP), although quinolones are no longer effective after a fast rise in resistance mechanisms among *Campylobacter* species (Carreira et al., 2012; Hormeño et al., 2016). Aminoglycosides, the third class of antimicrobials used worldwide after sulfonamides and beta-lactams, are a recommended alternative for the treatment of difficult infections caused by thermotolerant *Campylobacter* spp. (Wieczorek and Osek, 2013). The advantages of using aminoglycosides compared to other antimicrobials are their concentration-dependent bactericidal activity and relatively predictable pharmacokinetics, and synergism with other antibiotics (Vakulenko and Mobashery, 2003). Among aminoglycosides, the first active molecule used was streptomycin (STR), produced by *Streptomyces griseus*. STR binds to the aminoacyl-tRNA site (A site) of the 16S rRNA in the 30S ribosomal subunit, inducing codon misreading and inhibiting of translocation (Moazed and Noller, 1987; Woodcock et al., 1991) which leads to inadequate protein production. When antibiotic resistance appears it is due to target modification of ribosomal components, antimicrobial modification, or lowering drug accumulation in the cell (Vakulenko and Mobashery, 2003). Like in other bacteria, mutation K43R of S12 protein, a component of the 30S ribosomal subunit encoded by the *rpsL* gene, confers high-level of STR resistance in *Campylobacter* (Olkkola et al., 2010). Besides that, two out of four ANT(6)-I subfamily members of aminoglycoside nucleotidyltransferases (also known as aminoglycosides adenyltransferases of the AADE family), ANT(6)-Ia and ANT(6)-Ib, are frequently involved in STR resistance in *Campylobacter* strains and probably evolved from Gram-positive bacteria (Pinto-Alphandary et al., 1990; Shaw et al., 1993; Gibreel et al., 2004; Nirdnoy et al., 2005; Abril et al., 2010; Qin et al., 2012; Zhao et al., 2016). An additional role in STR resistance of ANT-like protein has been suggested in *C. coli* (Olkkola et al., 2016).

The aim of this work was to characterize the STR resistance presented in *Campylobacter* isolates of human and animal origin, establishing the role of a new enzyme of the ANT(6)-I family, ANT(6)-Ie, detected in a significant fraction of STR resistant isolates which molecular typing evidenced spread between animal and human hosts.

## Materials and Methods

### Bacteria and Antimicrobial Resistance

*Campylobacter* spp. strains isolated from humans were previously described (Hormeño et al., 2016) and resulted from systematical screenings performed during 2010–2012 in fecal samples from gastroenteritis patients by the Microbiology services of three hospitals located in West-Center Spain: San Pedro de Alcántara, Cáceres; Campo Arañuelo, Cáceres; and Universitario de Salamanca, Salamanca. *Campylobacter* spp. isolated from bovine, fattening pigs and poultry were randomly selected in 2010–2012 from slaughterhouses located all around Spain by the Spanish Surveillance Network of Antimicrobial Resistance in Bacteria of Veterinary Origin (VAV Network; Moreno et al., 2000) and were partially described elsewhere (Florez-Cuadrado et al., 2016). From each farm, a single *Campylobacter* isolate was obtained by culturing pooled feces from animals (bovine and porcine) and cloacal or meat samples (poultry). Isolates were grown on blood agar, in a microaerophilic atmosphere (CampyGenTM, Thermo Scientific) at 42°C for 24–48 h and were identified by a Vitek-MS MALDI-TOF system (bioMérieux, Marcy-l’Etoile, France) to species level. The minimal inhibitory concentrations (MICs) for STR, ERY, gentamicin (GEN), CIP, and tetracycline (TET) were determined by agar dilution methods according to the guidelines of CLSI (Clinical and Laboratory Standards Institute [CLSI], 2010), including *Campylobacter jejuni* ATCC 33560 as the reference strain. Resistance was determined according to the EUCAST ^2^ (last accessed September of 2018), by using cut-off values [ecological cut-off value (ECOFF)] of 4 mg/L for STR, 4 mg/L (*C. jejuni*) or 8 mg/L (*C. coli*) for ERY, 2 mg/L for GEN, 0.5 mg/L for CIP, and 1 mg/L (*C. jejuni*) or 2 mg/L (*C. coli*) for TET. To test the presence of efflux pumps, MIC to STR were determined in the presence of the efflux pump inhibitor phenylalanine-arginine beta-naphthylamide (PaβN, Sigma) at a concentration of 20 mg/L.

### Detection of Resistance Determinants

PCR was performed on DNA obtained by boiling, for 5 min, a suspension of one or two colonies from pure culture in 250 μL of milli-Q water, and recovering the supernatant after centrifugation at 10,000 ×*g* for 10 min. PCR was carried out with 1 μl of DNA, 0.2 mM of each dNTP (Biotools, Madrid, Spain), 0.5 μM of each primer [Stab Service (University of Extremadura, Badajoz, Spain)], 0.025 U/μl of Taq Polymerase (Biotools, Madrid, Spain) and 1X PCR buffer containing 1.5 mM MgCl_2_ (Biotools, Madrid, Spain), during 30 cycles of 94°C, 30 s; annealing temperature indicated in Table 1, 30 s; 72°C, 1 min. Amplicon purification was done with Speedtools PCR clean-up kit (Biotools, Madrid, Spain), following the manufacturer’s instructions. DNA sequencing were performed by STAB Service (DNA Sequencing facilities of the Universidad de Extremadura, Spain). *In silico* data analysis was carried out with bioinformatics tools available in NCBI^3^, SMS^4^, and EBI^5^.

**Table 1:** Primers used in this work.

Name	Sequence (5′-3′)	T^1^	Bp^2^	Reference
RPSLF	CCAGCGCTTAAAAAT TGTCC	55	247	Olkkola et al., 2010
RPSLR	TATCAAGAGCACCA CGAACG			
INT1F	GGCTCTCGGGTAAC ATCAAGG	54	242	Leverstein-van Hall et al., 2002
INT1R	TCAGGAGATCGGAA GACCTC			
CSF	GGCATCCAAGCAGCAAG	56	VAR^3^	Lévesque et al., 1995
CSR	AAAAGCAGACTTGA CCTGA			
SAF	TGCAAAA(G/A)CC(G/C) GA(A/G)GATATGG	56	305	This work
SAR	TTCCTT(G/T)CG(G/A) CATA(G/T)CC(C/T)TT			
SBF	GATTGT(T/C)CG(T/C)CAT GAGCTGCT	57	327	This work
SBR	GTGCTATCCAGGCAGC CGGTT			
SCF	TGCCT(A/C)AAATTGG(G/A) T(G/A)AGTT	52	368	This work
SCR	ACCTAGCCA(A/G)ATTTCA AA(A/G)CCAAA			
STREJF	TGCAAAGCGAAAA AAGAAT	49	878	This work
STREJR	TTATAATTTTCTTAAAAT TTTGCAAT			
STRECF	TGCAAAATCAAGATAAAT TTTTAAAAC	51	899	This work
STRECR	TTACAATTTTCCTAAAAT TTTACAAT			
STREFF	GTATGCGCAAAAATGAT TAAAG	50	1110	This work
STREFR	AAGGAAAAATTTAAATAT TGGTTTCA			


*^1^Annealing temperatures for PCR. ^*2*^PCR-Product size in bp. ^*3*^Variable size depending on gene-cassette structure (Lévesque et al., 1995).*

Mutations in the STR resistance region of the *rpsL* gene were screened by sequencing of the PCR amplicon produced by primers and conditions previously described (Table 1; Olkkola et al., 2010). Similarly, the possible presence of *ant(3”)-Ia* genes carried by Class-I integrons was evaluated by PCR with primers specific to *intI* and *intI*-associated gene cassettes (Table 1). Three sets of degenerated primers were designed to amplify internal fragments of genes *ant(6)-I* (Table 1): *ant(6)-Ia* (primers SAF and SAR), *ant(6)-Ib* (primers SBF and SBR), and *ant(6)-Ie* (primers SEF and SER). Further analysis was performed to amplify the (almost) full coding sequences of *ant(6)-Ie* genes (Table 1) from *C. jejuni* (primers STREJF and STREJR) and *C. coli* (primers STRECF and STRECR). Oligonucleotide design was performed with Oligo v.6 software.

### Functional Expression in *E. coli*

The expression of *ant(6)-Ie* from *C. coli* was tested through cloning the complete gene in the vector pGem-T Easy (Promega^®^), according to the manufacturer’s instructions. The full length of the gene including its promoter sequence was amplified by using primers STREFF and STREFR (Table 1), designed from the genome sequence of *C. coli* Z163 (ZP_14079546.1) and assuming that σ^70^ *Campylobacter* promoters have a well-conserved -10 box and lack the -35 box presented in other bacteria (Petersen et al., 2003). The ligation mixture was electroporated in *Escherichia coli* XL1-Blue MRF’ and transformants were selected in Luria-Bertani medium supplemented with 100 mg/L ampicillin.

### Multilocus Sequence Typing of *Campylobacter* Isolates From Human and Animal Origin

A group of *Campylobacter* isolates was genotyped for *fla*A-SVR (short variable region of *fla*A gene) and multilocus sequence typing (MLST). PCR fragments of the housekeeping genes *asp*A (aspartase A), *gln*A (glutamine synthetase), *glt*A (citrate synthase), *gly*A (serine hydroxymethyltransferase), *pgm* (phosphoglucomutase), *tkt* (transketolase), and *unc*A (ATP synthase a subunit), as well as *fla*A gene (flagellin), were amplified and sequenced as described elsewhere (Ugarte-Ruiz et al., 2013). Allele numbers were assigned by sequence comparisons against the existing sequences deposited in the *Campylobacter* MLST database^6^.

## Results

### Streptomycin Resistance Phenotypes in Isolates From Human Origin

**FIGURE 1:**
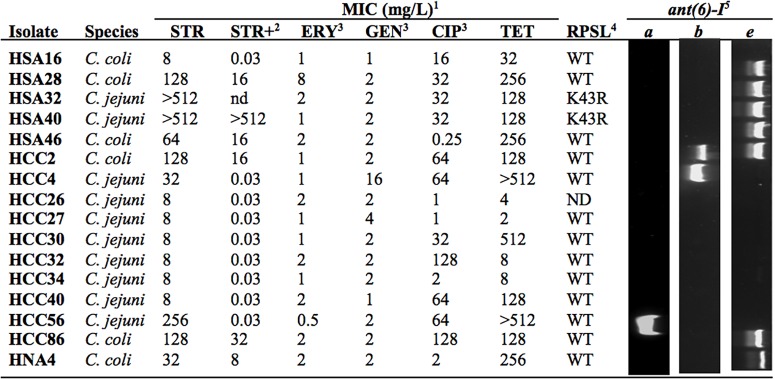
Phenotypic and genotypic analysis of streptomycin (STR) resistant isolates. ^1^Minimal inhibitory concentrations for STR, erythromycin (ERY), gentamicin (GEN), ciprofloxacin (CIP), and tetracycline (TET). ^2^MIC were determined in the presence of PaβN (mg/L). ^3^Data previously reported (Hormeño et al., 2016). ^4^Mutations in the RPSL coding sequence were detected by sequencing (WT, no mutation). ^5^Genes *ant(6)-I* were amplified with PCR with specific primers. ND, not determined.

Based on the ECOFF defined by EUCAST for STR resistance of *Campylobacter* (MIC > 4 mg/L), 16 out of 141 human isolates are above the threshold (Figure 1). Among these it was possible to identify three different phenotypes: high-level resistance, shown by two *C. jejuni* strains (MIC > 512 mg/L), medium-level resistance, in two *C. jejuni* and five *C. coli* isolates (32 ≤ MIC ≤ 256 mg/L), and low-level resistance, with inhibition of growth immediately above ECOFF, detected in six *C. jejuni* and one *C. coli* (MIC = 8 mg/L). Treatment with the efflux pump inhibitor PAβN reduced MICs in all the isolates, with the exception of the highly resistant HSA40, with maximal susceptibility attained in two isolates from the medium-level resistance group plus in the seven isolates with the lowest resistance level (Figure 1). Among analyzed isolates, low susceptibility against clinically relevant antimicrobials was generally found to CIP and/or TET but not to ERY or GEN, although three low-level resistant strains to STR were also found near the cut-off for CIP and TET (HCC26, HCC27, and HCC34; Figure 1).

### *rpsL* Polymorphism Among Streptomycin Resistant Isolates

The *rpsL* gene region determining resistance to aminoglycosides (Olkkola et al., 2010) was amplified and sequenced in 15 *Campylobacter* isolates with MICs above STR ECOFF value (Accession Nos. LT605180, LT605181, LT605182, LT605184, LT605185, LT605186, LT605187, LT605190, LT605191, LT605192, LT605193, LT605194, LT605195, LT605196, and LT605197). Among 11 polymorphic positions detected, only one was expressed at protein level corresponding to mutation K43R (not shown). This occurred in two *C. jejuni* isolates, HSA32 and HSA40 (Accession Nos. LT605194 and LT605195), having both the high-level resistant phenotype (Figure 1).

### The ANT(6)-I Family in *Campylobacter*

**FIGURE 2:**
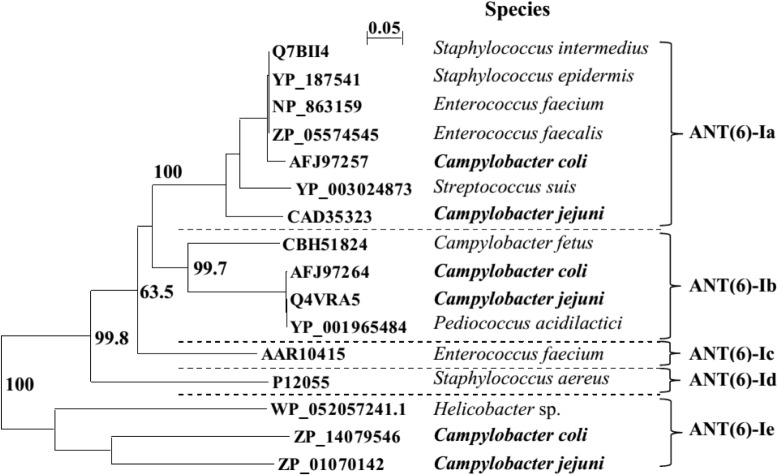
The ANT(6)-I phylogenetic tree. Multiple sequence alignment was performed by Clustal X 2.1. The phylogenetic tree was deduced by neighbor joining algorithm, excluding positions with gaps and emulated by NJPlot 2.3. Bootstrap values (N, 100; seeds, 111) are indicated for branches supporting sequence clustering and assuming previous data (Abril et al., 2010).

The NCBI database includes sequences for three members of the ANT(6) protein family previously described in *Campylobacter*: ANT(6)-Ia, ANT(6)-Ib, and ANT-like sequence cluster (Abril et al., 2010; Olkkola et al., 2016). The phylogenetic relationships previously defined within the ANT(6)-I family (Abril et al., 2010) were re-analyzed (Figure 2), including *C. jejuni* and *C. coli* for clusters ANT(6)-Ia and ANT(6)-Ib, plus the new and distantly related family member previously identified as ANT-like (Olkkola et al., 2016). Supported by bootstrapping with a threshold near 70%, ANT-like sequences cluster is a new member of the protein family that will be named hereafter ANT(6)-Ie (Figure 2), the fifth described ANT(6) (aminoglycoside 6-adenyltransferase) enzyme.

### ANT(6)-I Detection in Streptomycin Resistant Isolates

The role of ANT(6)-I enzymes on STR resistance of *Campylobacter* was addressed by using specific primers designed to detect the coding sequences for ANT(6)-Ia, ANT(6)-Ib, and ANT(6)-Ie, including degenerated positions for efficient amplification of homologs of either *C. jejuni* or *C. coli* for every subfamily (Table 1). Among the 16 *Campylobacter* isolates resistant to STR detected in this work from human infections, nine were positive for the presence of *ant(6)-I* genes with two isolates positive for the subfamilies *ant(6)-Ia*, one for *ant(6)-Ib* and seven for *ant(6)-Ie* (Figure 1). The unique two *C. jejuni* isolates presenting *ant(6)-Ie* also have the RSPL polymorphism K43R and the high-resistance phenotype, whereas the six isolates with low-level of resistance did not carry any of the screened genes.

The nucleotide sequences of the seven *ant(6)-Ie* genes detected among human isolates, including the six *Campylobacter* strains presenting this gene as the unique aminoglycoside 6-adenyltransferase enzyme, revealed different functional roles on STR resistance depending on *Campylobacter* species. The *ant(6)-Ie* genes from the two *C. jejuni* isolates were found non-functional when compared with the reference used to define the protein subfamily (ZP_01070142, Figure 2), sharing both the unique polymorphism C-394-Δ (Accession No. LT605198, isolate HSA32), an out of frame deletion that produces the premature arrest of translation and the loss of 55% of protein sequence from its C-terminal end. In contrast, the four *ant(6)-Ie* genes from *C. coli* strains HCC2, HSA28, HSA86, and HCC46 presented identical sequences to ZP_14079546.1, whereas the polymorphism C466T originating variant P156S in the encoded protein was detected in the gene from HNA4 isolate (Accession No. LT605200).

### Functional Expression in *E. coli* of ANT(6)-Ie

The coding sequence for ANT(6)-Ie from HNA4 was amplified and cloned in pGEM-T vector and *E. coli* XL1 Blue (MRF’) cells. Cells carrying the recombinant vector expressed resistance to STR with a MIC of 64 mg/L, significantly higher than the control cells transformed with a non-recombinant vector (MIC = 8 mg/L). Besides, both recipient and transformants cells remained sensitive to other antimicrobials tested showing aminoglycoside specificity of the *ant(6)-Ie* gene: spectinomycin (MIC ≤ 32 mg/L), GEN (MIC ≤ 1 mg/L), apramycin (MIC ≤ 4 mg/L), and neomycin (MIC ≤ 4 mg/L).

### Genetic and Phenotype Relationships Among Human and Animal Streptomycin Resistant Isolates Carrying ANT(6)-Ie

We screened for the three ANT(6)-I encoding genes in *Campylobacter* among 65 STR resistant isolates from the three most common food-producing livestock: poultry, pigs, and cattle (Table 2). All *ant(6)-I* genotypes were detected, with *C. coli* being largely the most prevalent species among streptomycin resistant isolates. Interestingly, the presence of the single-gene *ant(6)-Ie* genotype represents a major fraction of STR resistant *C. coli*, with one fourth of isolates.

**Table 2:** *ant(6)-I* genotypes of streptomycin resistant *Campylobacter* isolates.

Host	*ant(6)-I* profile^1^								
	a	b	e	a/b	a/e	b/e	a/b/e	Ø	Σ
Human^2^	1	1	6(4)	-	-	1(1)	-	7(1)	16 (6)
Poultry	13(8)	1(1)	-	10(9)	1(1)	-	2(1)	5(1)	32 (21)
Porcine	4(4)	-	10(10)	2(2)	7(7)	4(4)	1(1)	1(1)	29 (29)
Bovine	2(1)	-	1(1)	-	1(1)	-	-	-	4 (3)
Σ	20(13)	2(1)	17(15)	12(11)	9(9)	5(5)	3(2)	6(3)	81 (59)


*^1^Genotypes were deduced by PCR with primers SAF/R, SBF/R and SCF/R (Table 1). ^*2*^Genotypes of human isolates are shown in Figure 1. Data in parentheses refer to number of isolates belonging to *C. coli* species. Ø, zero genes detected.*

Multilocus sequence typing plus *flaA* typing was performed in 14 *C. coli* isolates carrying *ant(6)-Ie* as the only determinant expressing STR resistance (Table 3). The multilocus analysis allowed the detection of a cluster of strains (ST-827, clonal complex 828) including two isolates from human origin plus one from bovine. Moreover, one of the human and the bovine origin isolates shared the same *flaA* allele 236 and the same resistance profile against the five clinically relevant antimicrobials tested, which is considered an indication of a probable common clonal origin.

**Table 3:** Molecular and antimicrobial resistance typing of *Campylobacter* isolates carrying^1^ *ant(6)-Ie*.

			MIC (mg/L)^2^							
Strain	Year	Origin	STR	ERY	GEN	CIP	TET	CC^3^	ST^4^	*flaA*
ZTA10/00526CPD	2010	Porcine	≥32	1	4	≥8	≥32	ST-828	7337	ND
ZTA10/00602CPD	2010	Porcine	≥32	≥64	4	≥8	≥32	ND	7340	ND
ZTA10/00794CPD	2010	Porcine	≥32	1	4	≥8	≥32	ST-828	829	ND
ZTA10/01257CPD	2010	Bovine	≥32	1	2	≥8	≥32	ST-828	827	0236
ZTA10/01418CPD	2010	Porcine	≥32	≥64	2	≥8	≥32	ST-828	1413	ND
ZTA10/02049CPD	2010	Porcine	≥32	2	2	≥8	≥32	ST-828	4950	ND
ZTA11/00514CP	2011	Porcine	≥32	≥64	2	≥8	≥32	ND	7341	0662
ZTA11/00726CP	2011	Porcine	≥32	1	4	0.13	≥32	ST-828	7338	ND
ZTA11/01342CP	2011	Porcine	≥32	≥64	4	0.25	≥32	ST-828	1413	ND
ZTA11/03282CP	2011	Porcine	≥32	0.5	1	≥8	≥32	ST-828	1096	0319
ZTA11/03389CP	2011	Porcine	≥32	≥64	2	≥8	≥32	ST-828	2733	ND
HSA028	2010	Human	128	8	2	32	256	ST-828	827	0236
HSA046	2010	Human	64	2	2	0.25	256	ST-828	827	0255
HNA4	2010	Human	32	2	2	2	256	ND	7339	0633


*^1^The fourteen *C. coli* isolates presenting *ant(6)-Ie* as the unique streptomycin (STR) resistance determinant (Table 2). ^*2*^Minimal inhibitory concentrations for STR, erythromycin (ERY), gentamicin (GEN), ciprofloxacin (CIP), and tetracycline (TET). ^*3*^Clonal Complex. ^*4*^Sequence Types and *flaA* alleles were assigned by MLST database (see footnote 6). ND, not determined.*

## Discussion

This work shows the main role of adenylyl transferases belonging to the ANT(6)-I family on STR resistance in *Campylobacter*. Previous reports had described the phenotypic expression of ANT(6)-I enzymes (Nirdnoy et al., 2005; Abril et al., 2010; Qin et al., 2012; Olkkola et al., 2016), and now strong evidence is provided supporting the role of ANT(6)-Ie on STR resistance. Although ANT(6)-Ie coding sequences were detected in the two most frequent *Campylobacter* species, *C. jejuni* and *C. coli*, the association with STR resistance was only proved in *C. coli* since no *C. jejuni* isolate carried this coding sequence as the unique candidate to express the phenotype (Figure 1 and Table 2).

Besides ANT(6)-I, an additional STR resistance determinant is ANT(3”)-Ia or AADA which also confers resistance to spectinomycin. This enzyme is highly prevalent among enterobacteria (Shaw et al., 1993) and has been detected associated to class I integrons and their gene cassettes in *Campylobacter*, although only anecdotally (Ouellette et al., 1987; O’Halloran et al., 2004). Indeed, several reports have described the unsuccessful search of *ant(3”)* in *Campylobacter* (van Essen-Zandbergen et al., 2007; Piccirillo et al., 2013). Similarly, all STR resistant isolates from humans analyzed in the present work have been screened for *int1* or associated gene cassettes, unsuccessfully (data not shown). Thus, ANT(6)-I enzymes might be the unique adenylyl transferases with significant relevance in STR resistance in *Campylobacter*.

To the best of our knowledge, this is the first report showing a RPSL mutation in *C. jejuni* isolates conferring STR resistance. In a previous study, with *C. coli*, it was found that isolates presenting high-level resistance to STR shared the mutation K43R in RPSL (Olkkola et al., 2010), similarly to the two *C. jejuni* isolates from humans, detected in this work, with MIC > 512 mg/L (Figure 1). Although both isolates also carry *ant(6)-Ie* genes, resistance to STR might be determined by RPSL mutation since the adenylyl transferase coding sequence is truncated and most probably not functional. In addition, there was no contribution to this phenotype from efflux pump activity, as deduced by the lack of any effect on MIC by PAβN treatment (Figure 1).

A group of six *C. jejuni* and one *C. coli* isolates from humans that expressed low-level STR-resistance, did not contain any of the screened determinants and presented a strong decreased MIC to STR in the presence of PAβN (Figure 1). Thus, efflux pump activity must be responsible for low-level STR resistance of these strains, similarly to *Mycobacterium tuberculosis* where the effect of outward transporters is known to increase modestly the MIC for STR (Spies et al., 2008). At least three different efflux pump systems have been shown to be up-regulated in *Campylobacter* strains resistant to a broad range of antimicrobials (Lin et al., 2005; Akiba et al., 2006; Jeon et al., 2011), so they could be candidates for determinants to the low level STR resistance. In addition, treatment with PAβN produced a strong effect on MIC of *Campylobacter* isolates carrying *ant(6)-I* genes, mostly for those with *ant(6)-Ia* or *ant(6)-Ib* as unique resistance determinants (Figure 1). This observation might indicate that, among human isolates analyzed in this work, the only functional adenylyl transferase gene is *ant(6)-Ie* and that even these isolates require efflux pump activity to support the medium-level of resistance. Treatment of *ant(6)-Ie* carrying strains with PAβN reduces their STR MIC to low-level resistance, which might correspond to their *in vivo* expression level. Synergic effects of efflux pumps have been evidenced in *Campylobacter* with resistance determinants for quinolones and macrolides, *gyr*A and 23S rRNA gene mutations, respectively (Luo et al., 2003; Cagliero et al., 2006; Corcoran et al., 2006). Indeed, three *Campylobacter* isolates showing low-level resistance to STR were also found to have low-level resistant to CIP and TET (Figure 1), lacked the *gyrA* C-257-T mutation conferring low susceptibility to fluoroquinolones (Hormeño et al., 2016) and also *tetO*, the major TET resistant determinant in this species (not shown, authors’ personal communication). A weak overexpression of efflux pump activity might be involved in the antimicrobial resistance phenotype of these strains.

The set of primers described in this work allows specific detection of the three *ant(6)-I* genes described in *Campylobacter*, including those belonging to *ant(6)-Ie* and encoding a new subfamily of aminoglycoside O-nucleotidyltransferases (Figure 2) that provides functional information for hundreds of orthologs annotated as hypothetical proteins, mainly from *Campylobacter* and related organisms like *Helicobacter*. In addition, the molecular and antimicrobial resistance typing of *Campylobacter* isolates expressing ANT(6)-Ie has revealed a spread pathway for this zoonotic pathogen between cattle and humans.

## Author Contributions

SP and AQ conceived and designed the study. LH, MU-R, GP, CB, and DF-C acquired the samples and data. LH, MU-R, GP, DF-C, and MC performed the laboratory analysis. SV, SP, LD, MC, and AQ analyzed and interpreted the data. MC and AQ wrote the manuscript. All authors have approved the final article.

## Conflict of Interest Statement

The authors declare that the research was conducted in the absence of any commercial or financial relationships that could be construed as a potential conflict of interest.

## Footnotes

1 www.fda.gov/AnimalVeterinary/SafetyHealth/AntimicrobialResistance/NationalAntimicrobialResistanceMonitoringSystem/default.htm

2 www.eucast.org

3 http://www.ncbi.nlm.nih.gov

4 http://bioinformatics.org/sms

5 http://www.ebi.ac.uk

6 http://pubmlst.org/campylobacter

## References

[B1] Abril C. Brodard I. Perreten V. (2010). Two novel antibiotic resistance genes, *tet(44)* and *ant(6)-Ib*, are located within a transferable athogenicity island in *Campylobacter fetus* subsp *fetus*. *Antimicrob. Agents Chemother.* 54 3052–3055. 10.1128/AAC.00304-10 20479200 PMC2897286

[B2] Akiba M. Lin J. Barton Y. W. Zhang Q. J. (2006). Interaction of CmeABC and CmeDEF in conferring antimicrobial resistance and maintaining cell viability in *Campylobacter jejuni*. *J. Antimicrob. Chemother.* 57 52–60. 10.1093/jac/dki419 16303882

[B3] Cagliero C. Mouline C. Cloeckaert A. Payot S. (2006). Synergy between efflux pump CmeABC and modifications in ribosomal proteins L4 and L22 in conferring macrolide resistance in *Campylobacter jejuni* and *Campylobacter coli*. *Antimicrob. Agents Chemother.* 50 3893–3896. 10.1128/AAC.00616-06 16940070 PMC1635205

[B4] Carreira A. C. Clemente L. Rocha T. Tavares A. Geraldes M. Barahona M. J. (2012). Comparative genotypic and antimicrobial susceptibility analysis of zoonotic Campylobacter species isolated from broilers in a nationwide survey Portugal. *J. Food Prot.* 75 2100–2109. 10.4315/0362-028X.JFP-12-183 23212005

[B5] Clinical and Laboratory Standards Institute [CLSI] (2010). *Methods for Antimicrobial Dilution and Disk Susceptibility Testing for Infrequently-Isolated or Fastidious Bacteria: Approved Guidelines Approved Guidelines (M45-A)*. Wayne, PA: CLSI.

[B6] Collective Eurosurveillance Editorial Team (2015). The 2013 joint ECDC/EFSA report on trends and sources of zoonoses, zoonotic agents and food-borne outbreaks published. *Euro Surveill.* 20:21021. 10.2807/ese.20.04.21021-en 25655057

[B7] Corcoran D. Quinn T. Cotter L. Fanning S. (2006). An investigation of the molecular mechanisms contributing to high-level erythromycin resistance in *Campylobacter*. *Int. J. Antimicrob. Agents* 27 40–45. 10.1016/j.ijantimicag.2005.08.019 16318913

[B8] Florez-Cuadrado D. Ugarte-Ruiz M. Quesada A. Palomo G. Domínguez L. Porrero M. C. (2016). Description of an erm(B)-carrying *Campylobacter coli* isolate in Europe. *J. Antimicrob. Chemother.* 71 841–843. 10.1093/jac/dkv383 26604242

[B9] Gibreel A. Skold O. Taylor D. E. (2004). Characterization of plasmid-mediated aphA-3 kanamycin resistance in *Campylobacter jejuni*. *Microb. Drug Resist.* 10 98–105. 10.1089/1076629041310127 15256024

[B10] Hormeño L. Palomo G. Ugarte-Ruiz M. Porrero M. C. Borge C. Vadillo S. (2016). Identification of the main quinolone resistance determinant in *Campylobacter jejuni* and *Campylobacter coli* by MAMA-DEG PCR. *Diagn. Microbiol. Infect. Dis.* 84 236–239. 10.1016/j.diagmicrobio.2015.11.002 26658311

[B11] Jeon B. Wang Y. Hao H. Barton Y.-W. Zhang Q. (2011). Contribution of CmeG to antibiotic and oxidative stress resistance in *Campylobacter jejuni*. *J. Antimicrob. Chemother.* 66 79–85. 10.1093/jac/dkq418 21081547 PMC3001851

[B12] Leverstein-van Hall M. A. Paauw A. Box A. T. A. Blok H. E. M. Verhoef J. Fluit A. C. (2002). Presence of integron-associated resistance in the community is widespread and contributes to multidrug resistance in the hospital. *J. Clin. Microbiol.* 40 3038–3040. 10.1128/JCM.40.8.3038-3040.2002 12149373 PMC120645

[B13] Lévesque C. Piché L. Larose C. Roy P. H. (1995). PCR mapping of integrons reveals several novel combinations of resistance genes. *Antimicrob. Agents Chemother.* 39 185–191. 7695304 10.1128/aac.39.1.185 PMC162507

[B14] Lin J. Akiba M. Sahin O. Zhang Q. J. (2005). CmeR functions as a transcriptional repressor for the multidrug efflux pump CmeABC in *Campylobacter jejuni*. *Antimicrob. Agents Chemother.* 49 1067–1075. 10.1128/AAC.49.3.1067-1075.2005 15728904 PMC549222

[B15] Luo N. Sahin O. Lin J. Michel L. O. Zhang Q. J. (2003). In vivo selection of *Campylobacter* isolates with high levels of fluoroquinolone resistance associated with gyrA mutations and the function of the CmeABC efflux pump. *Antimicrob. Agents Chemother.* 47 390–394. 10.1028/AAC.47.1.390-394.2003 12499221 PMC148968

[B16] Moazed D. Noller H. F. (1987). Interaction of antibiotics with functional sites in 16S ribosomal RNA. *Nature* 327 389–394. 10.1038/327389a0 2953976

[B17] Moreno M. A. Domínguez L. Teshager T. Herrero I. A. Porrero M. C. (2000). Antibiotic resistance monitoring: the Spanish programme. The VAV Network. Red de Vigilancia de Resistencias Antibióticas en Bacterias de Origen Veterinario. *Int. J. Antimicrob. Agents* 14 285–290. 10.1016/S0924-8579(00)00138-2 10794948

[B18] Nirdnoy W. Mason C. J. Guerry P. (2005). Mosaic structure of a multiple-drug-resistant, conjugative plasmid from *Campylobacter jejuni*. *Antimicrob. Agents Chemother.* 49 2454–2459. 10.1128/AAC.49.6.2454-2459.2005 15917546 PMC1140535

[B19] O’Halloran F. Lucey B. Cryan B. Buckley T. Fanning S. (2004). Molecular characterization of class 1 integrons from Irish thermophilic *Campylobacter spp*. *J. Antimicrob. Chemother.* 53 952–957. 10.1093/jac/dkh193 15128721

[B20] Olkkola S. Culebro A. Juntunen P. Hanninen M.-L. Rossi M. (2016). Functional genomics in Campylobacter coli identified a novel streptomycin resistance gene located in a hypervariable genomic region. *Microbiology* 162 1157–1166. 10.1099/mic.0.000304 27154456

[B21] Olkkola S. Juntunen P. Heiska H. Hyytiainen H. Hanninen M. L. (2010). Mutations in the rpsL gene are involved in streptomycin resistance in *Campylobacter coli*. *Microb. Drug Resist.* 16 105–110. 10.1089/mdr.2009.0128 20370506

[B22] Ouellette M. Gerbaud G. Lambert T. Courvalin P. (1987). Acquisition by a Campylobacter-like strain of aphA-1, a kanamycin resistance determinant from members of the family *Enterobacteriaceae*. *Antimicrob. Agents Chemother.* 31 1021–1026. 10.1128/AAC.31.7.1021 2821885 PMC174865

[B23] Petersen L. Larsen T. S. Ussery D. W. On S. L. W. Krogh A. (2003). RpoD promoters in *Campylobacter jejuni* exhibit a strong periodic signal instead of a-35 box. *J. Mol. Biol.* 326 1361–1372. 10.1016/S0022-2836(03)00034-2 12595250

[B24] Piccirillo A. Dotto G. Salata C. Giacomelli M. (2013). Absence of class 1 and class 2 integrons among *Campylobacter jejuni* and *Campylobacter coli* isolated from poultry in Italy. *J. Antimicrob. Chemother.* 68 2683–2685. 10.1093/jac/dkt242 23788478

[B25] Pinto-Alphandary H. Mabilat C. Courvalin P. (1990). Emergence of aminoglycoside resistance genes aadA and aadE in the genus *Campylobacter*. *Antimicrob. Agents Chemother.* 34 1294–1296. 10.1128/AAC.34.6.1294 2168151 PMC171807

[B26] Qin S. Wang Y. Zhang Q. Chen X. Shen Z. Deng F. (2012). Identification of a novel genomic island conferring resistance to multiple aminoglycoside antibiotics in *Campylobacter coli*. *Antimicrob. Agents Chemother.* 56 5332–5339. 10.1128/AAC.00809-12 22869568 PMC3457361

[B27] Shaw K. J. Rather P. N. Hare R. S. Miller G. H. (1993). Molecular genetics of aminoglycoside resistance genes and familial relationships of the aminoglycoside-modifying enzymes. *Microbiol. Rev.* 57 138–163. 8385262 10.1128/mr.57.1.138-163.1993 PMC372903

[B28] Spies F. S. da Silva P. E. A. Ribeiro M. O. Rossetti M. L. Zaha A. (2008). Identification of mutations related to streptomycin resistance in clinical isolates of *Mycobacterium tuberculosis* and possible involvement of efflux mechanism. *Antimicrob. Agents Chemother.* 52 2947–2949. 10.1128/AAC.01570-07 18541729 PMC2493096

[B29] Ugarte-Ruiz M. Wassenaar T. M. Gomez-Barrero S. Porrero M. C. Navarro-Gonzalez N. Dominguez L. (2013). The effect of different isolation protocols on detection and molecular characterization of *Campylobacter* from poultry. *Lett. Appl. Microbiol.* 57 427–435. 10.1111/lam.12130 23837671

[B30] Vakulenko S. B. Mobashery S. (2003). Versatility of aminoglycosides and prospects for their future. *Clin. Microbiol. Rev.* 16 430–450. 10.1128/CMR.16.3.430-450.2003 12857776 PMC164221

[B31] van Essen-Zandbergen A. Smith H. Veldman K. Mevius D. (2007). Occurrence and characteristics of class 1, 2 and 3 integrons in *Escherichia coli*, *Salmonella* and *Campylobacter spp*. in the Netherlands. *J. Antimicrob. Chemother.* 59 746–750. 10.1093/jac/dkl549 17307772

[B32] Wieczorek K. Osek J. (2013). Antimicrobial resistance mechanisms among Campylobacter. *Biomed. Res. Int.* 2013:340605 10.1155/2013/340605 PMC3707206 23865047

[B33] Woodcock J. Moazed D. Cannon M. Davies J. Noller H. F. (1991). Interaction of antibiotics with A- and P-site-specific bases in 16S ribosomal RNA. *EMBO J.* 10 3099–3103. 1915283 10.1002/j.1460-2075.1991.tb07863.x PMC453027

[B34] Zhao S. Tyson G. H. Chen Y. Li C. Mukherjee S. Young S. (2016). Whole-genome sequencing analysis accurately predicts antimicrobial resistance phenotypes in *Campylobacter spp*. *Appl. Environ. Microbiol.* 82 459–466. 10.1128/AEM.02873-15 26519386 PMC4711122

